# Sociodemographic Predictors of Postpresentation Delay to Pediatric ACL Reconstruction and Associated Intra-articular Injury Severity

**DOI:** 10.1177/03635465261469687

**Published:** 2026-08-03

**Authors:** Christopher D. Hamad, Shannon Wu, Kole Joachim, Esther Chung, Timothy Liu, Mauricio Silva, Richard Bowen, Soroush Baghdadi

**Affiliations:** * Department of Orthopaedic Surgery, University of California, Los Angeles, California, USA; † David Geffen School of Medicine at UCLA, Los Angeles, California, USA; ‡ University of California, Los Angeles, Los Angeles, California, USA; § Luskin Orthopaedic Institute for Children, Los Angeles, California, USA; Investigation performed at University of California, Los Angeles, Los Angeles, California, USA

**Keywords:** pediatric anterior cruciate ligament, health disparities, Childhood Opportunity Index, return to sport, cartilage injury, access to care

## Abstract

**Background:**

Delayed anterior cruciate ligament reconstruction (ACLR) in pediatric patients is associated with progressive intra-articular injury. Although disparities in access to orthopaedic care have been reported, it remains unclear whether structural determinants of health influence postpresentation surgical delay and downstream functional recovery.

**Purpose:**

To determine whether sociodemographic and structural determinants are associated with prolonged postpresentation delay to pediatric ACLR and with intra-articular injury severity and postoperative functional outcomes.

**Study Design:**

Cohort study; Level of evidence, 3.

**Methods:**

The records of 443 pediatric patients who underwent ACLR (2013-2025) at an urban academic referral center with a pediatric orthopaedic urgent care were reviewed. Exposures included race/ethnicity, insurance type, and neighborhood deprivation (Childhood Opportunity Index [COI]). Delay was defined as time from first orthopaedic presentation to surgery, with prolonged delay defined as >120 days. Outcomes included high-grade cartilage injury (Outerbridge grade 3 or 4), meniscal tear presence, failure to return to sport (≥365 days of follow-up), hop test symmetry, isometric strength symmetry, and graft failure. Multivariable regression models were adjusted for race/ethnicity, age, sex, insurance, and national COI.

**Results:**

The median time from injury to presentation was 5 days, while the median postpresentation time to surgery was 101 days; 38.6% experienced prolonged delay. In adjusted logistic regression, non-Hispanic Black, Hispanic, and other/unknown race/ethnicity; public insurance; and lower neighborhood opportunity were each independently associated with prolonged delay. Continuous postpresentation delay was independently associated with increased odds of high-grade cartilage injury (OR, 1.004 per day; 95% CI, 1.000-1.008; *P* = .048). Among patients with ≥365 days of follow-up (n = 228), public insurance was not independently associated with failure to return to sport (OR, 1.69; *P* = .398). Among those completing functional testing (n = 150), public insurance (β = +9.14; *P* = .046), higher national COI (β = +0.14 per unit; *P* = .045), and male sex (β = +9.59; *P* = .010) were each independently associated with greater hop test asymmetry, whereas no independent associations were observed for isometric strength symmetry or graft failure.

**Conclusion:**

Disparities in pediatric ACLR are driven primarily by postpresentation system-level delays and are associated with an incrementally greater risk of cartilage injury.

Pediatric anterior cruciate ligament (ACL) injuries are increasingly common, with an incidence exceeding that of adult injuries.^3^ This increase may be explained by increased participation in organized sports, early specialization, and year-round training. Subsequently, pediatric ACL reconstruction (ACLR) has become an increasingly common surgical procedure, with an incidence rate of 50.9 per 100,000 children, a 5.7-fold increase in the last 2 decades.^6,18^ As the prevalence of ACL injuries increases, the disparities in access to treatment and consequent outcomes grow more apparent.

Prior research has shown that increased time from ACL injury to surgical management is associated with increased rates of chondral and meniscal injuries.^11,12,16^ Lawrence et al^11^ specifically found that pediatric patients with ACL injuries who experience >12 weeks of delay in surgery are more likely to present with chondral and meniscal injuries, including irreparable medial meniscus tears. These concomitant injuries may lead to suboptimal postoperative outcomes, delayed return to activities, and eventually long-term pain and arthritis.^11^ The reasons for these disparities are multifactorial; barriers to care may be influenced by socioeconomic status, insurance, or health literacy. For example, pediatric patients with private insurance are significantly more likely to obtain an appointment with an orthopaedic sports surgeon within 2 weeks of injury than those with public insurance.^14^ Similarly, Williams et al^19^ found that patients with public insurance experienced a delay in evaluation approximately twice as long as those with private insurance.

Our facility is an urban academic quaternary referral center for pediatric orthopaedics with a stand-alone pediatric orthopaedic urgent care specializing in musculoskeletal injuries. This care model provides rapid initial orthopaedic evaluation regardless of insurance status and serves a predominantly publicly insured and racially/ethnically diverse patient population, creating a unique opportunity to isolate postpresentation system-level delays from injury-to-presentation delays that have been the focus of prior work. Critically, prior studies examining disparities in ACL care have not controlled for access to initial orthopaedic evaluation, conflating delays attributable to barriers in reaching a provider with those arising after diagnosis. By ensuring uniform access to timely initial evaluation across all payer types, our care model allows us to identify the postpresentation interval as the primary focus of disparity and better isolate structural determinants that drive delays in definitive surgical management. Using this framework, we aimed to (1) determine whether sociodemographic and structural determinants of health are associated with delays in operative management of pediatric ACL injuries within the postpresentation interval, and (2) assess whether these delays are associated with increased intra-articular injury severity at the time of surgery.

## Methods

### Study Design and Setting

We conducted a retrospective, single-institution cohort study for all pediatric ACL injuries from April 2013 to June 2025. Patients were included if they were aged ≤18 years at the time of surgery, had a confirmed ACL tear, and underwent primary ACLR at our institution during the study period. Of 461 eligible patients identified, 18 were excluded: 10 due to use of a nonstandard graft type (anterior tibialis or iliotibial band allograft) and 8 due to unverifiable date of injury yielding a final analytic cohort of 443 patients. The primary objective was to determine whether sociodemographic and structural determinants (race/ethnicity, insurance type, and neighborhood deprivation measured by the national Childhood Opportunity Index [COI] 3.0) were associated with delays in operative care, and to assess whether prolonged delay was associated with greater intra-articular pathology at the time of surgery. Uniquely, this cohort allowed isolation of postpresentation system-level delay from injury-to-presentation delay, leveraging a care model in which initial orthopaedic evaluation occurs rapidly through a dedicated pediatric orthopaedic urgent care.

### Primary Exposure

The primary exposures were race/ethnicity (categorized into non-Hispanic White, non-Hispanic Black, Hispanic, and other/unknown), insurance type (private and public insurance including Medicaid/Medi-Cal), and neighborhood-level deprivation. The national COI 3.0 percentile score served as the primary neighborhood deprivation measure for all analyses. The COI is a composite index of child-specific neighborhood opportunity, integrating educational, health/environmental, and social/economic domains.^13^ In sensitivity analyses of the continuous delay outcomes (time from injury to presentation and time from presentation to surgery), the Area Deprivation Index (ADI) state decile and the health/environment subdomain of the COI were analyzed to assess the impact of neighborhood deprivation on the primary outcome across multiple measures.^10^ Patient age at surgery and sex were included as demographic covariates and utilized for adjustment in final regression models.

### Time-to-Care and Intra-articular Pathology Outcomes

Two complementary delay intervals were evaluated. First, overall delay was defined as time from the reported date of injury to the date of surgery. Second, to isolate system-level delays after presentation, we defined postpresentation delay as time from the first orthopaedic presentation within our pediatric urgent care, most commonly, to the date of surgery. To examine whether delay was associated with intra-articular damage at the time of surgery, cartilage and meniscal pathology were assessed. Cartilage injury was graded using the Outerbridge classification as documented in the operative report at the time of arthroscopic assessment. Cartilage injury was examined using low-grade versus high-grade injury (grades 0 and 2 vs grades 3 and 4).^7^ Meniscal pathology was analyzed as both the presence of any meniscal tear and the tear distribution pattern (none, medial only, lateral only, or both). For cartilage and meniscal outcomes, multivariable logistic regression was used, with meniscal injury modeled as a binary outcome (any tear vs none). Delay was operationalized using both a prolonged delay indicator (>120 days) and continuous log-transformed delay sensitivity analyses. The 120-day threshold was selected to approximate the median postpresentation delay in this cohort (101 days; IQR, 63-149 days), falling within the interquartile range of observed delays and providing a fixed, externally applicable definition of prolonged delay rather than a sample-specific median split.

### Return-to-Sport, Functional Performance, and Graft Failure Outcomes

In secondary analyses, we evaluated return-to-sport participation, functional performance, and graft failure among patients with at least 365 days of postoperative follow-up to allow adequate recovery time. Return to sport or desired activities was abstracted from the medical record and modeled as a binary outcome. Return to sport was defined as documented clearance by the treating surgeon to return to full sport participation without restriction, with no documentation of complaints regarding pain or instability at subsequent follow-up precluding participation in desired activities or sport. Postoperative follow-up at our institution routinely includes visits at 10 to 14 days, 6 weeks, 3 months, 6 months, 9 months, and 12 months after ACLR, with return-to-sport functional testing including hop test and isometric strength symmetry performed at the 7- to 9-month visit or later, reflecting variability in rehabilitation progression, scheduling, and individual recovery trajectories. A minimum of 365 days of total follow-up was required for inclusion in all secondary analyses, ensuring that patients had sufficient opportunity to complete rehabilitation, undergo functional testing, and receive clearance for unrestricted sport participation, and to confirm return-to-sport status and recovery trajectory beyond the testing visit itself. Functional testing data including hop test and isometric strength symmetry therefore reflect assessments performed at the 7- to 9-month visit among patients confirmed to have completed at least 365 days of follow-up. For patients cleared to return, subsequent follow-up was performed on an as-needed basis. Return-to-sport status was determined from the most recent clinical encounter documenting sport participation status. Among patients with documented return-to-sport functional testing, dynamic limb symmetry was assessed using a hop test composite calculated as the mean side-to-side difference across 4 standardized functional tests: single-leg hop for distance, triple hop for distance, 6-m timed hop, and crossover hop. Isometric strength symmetry was similarly evaluated using a composite calculated as the mean side-to-side difference across 5 strength assessments, including hip abduction, hip adduction, hip extension, quadriceps strength, and hamstring strength. For each component measure, symmetry was defined as the difference between operative and contralateral limbs, and more negative values reflect worse operative limb performance. Graft failure was evaluated as a secondary outcome and defined as clinical or MRI-confirmed complete graft rerupture or persistent graft insufficiency requiring revision ACLR. Multivariable models for all outcomes were adjusted for race/ethnicity, age at surgery, sex, insurance type, and national COI.

### Statistical Analysis

Continuous variables are summarized using medians with interquartile ranges, and categorical variables are summarized using counts with percentages. Unadjusted comparisons of delay distributions across race/ethnicity and other sociodemographic groups were performed using the Kruskal-Wallis rank-sum test. Associations between neighborhood socioeconomic context and delay were assessed using Spearman rank correlation. For unadjusted comparisons of categorical injury outcomes across race/ethnicity, Pearson chi-square tests were used. To evaluate drivers of delay, multivariable linear regression was used with log-transformed delay as the dependent variable. Regression coefficients (β) are reported with 95% confidence intervals, and exponentiated coefficients can be interpreted as the multiplicative change in delay per unit increase in the predictor. To evaluate associations between prolonged delay as well as delay and intra-articular pathology, return to sport, and graft failure, multivariable logistic regression was used, with results reported as odds ratios with 95% confidence intervals. Meniscal tear pattern (none, medial only, lateral only, or both) was modeled using multinomial logistic regression with “no meniscal tear” as the reference outcome, with results reported as odds ratios with 95% confidence intervals. Delay was operationalized using both a prolonged delay threshold (>120 days) and continuous log-time sensitivity analyses. All models were adjusted for race/ethnicity, age at surgery, sex, neighborhood deprivation (national COI in primary analyses; ADI and health COI in sensitivity analyses for the 2 continuous delay outcomes), and insurance type, with non-Hispanic White race/ethnicity, female sex, and private insurance as reference categories. Statistical significance was assessed using 2-sided testing with significance set at a *P* value <.05.

## Results

### Cohort Characteristics

**Figure 1.**
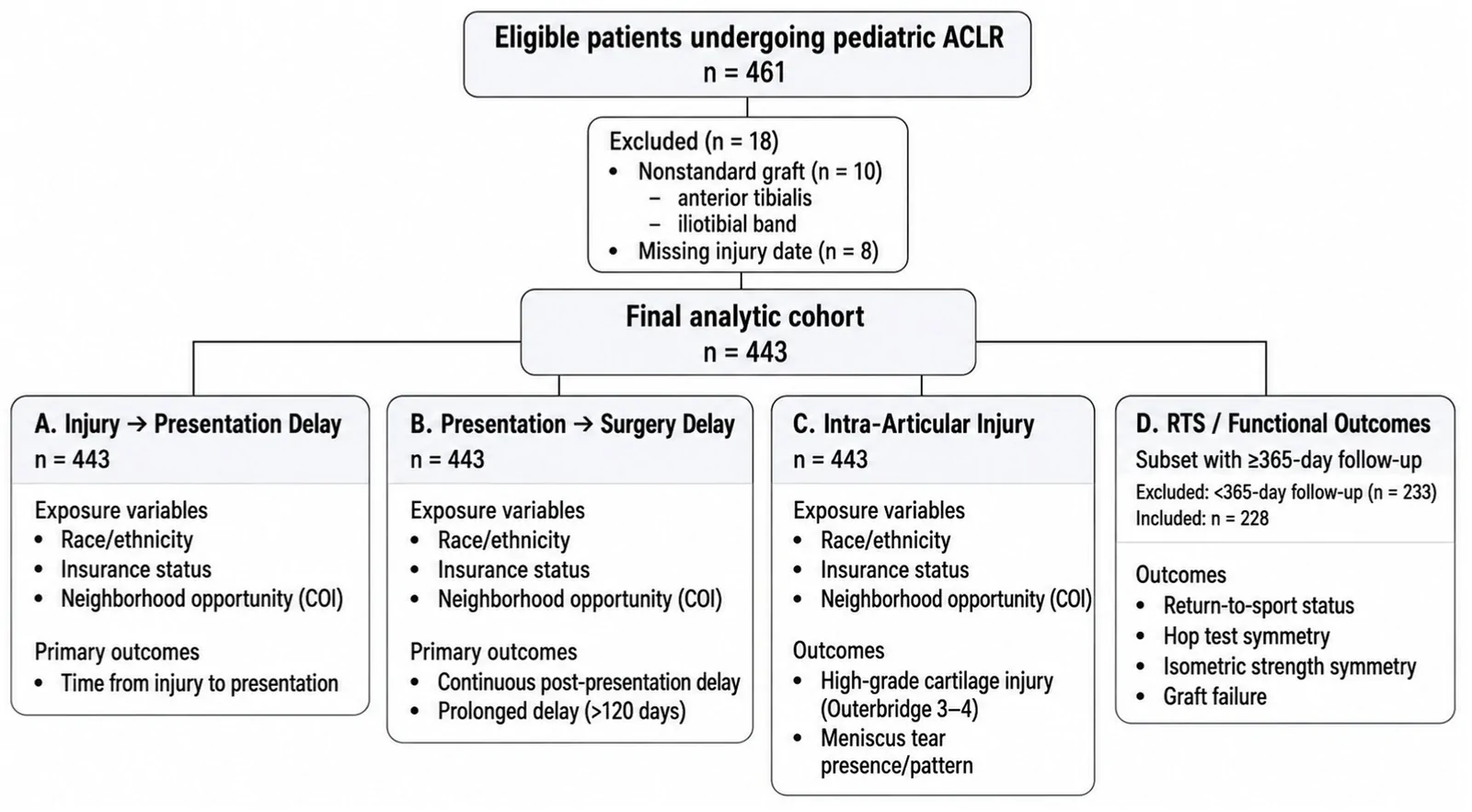
Study flow and analytic framework. Flow diagram illustrating cohort selection and the 4 primary analytic domains evaluated, including delay-related analyses and intra-articular injury assessments.

The final analytic cohort included 443 patients (Table 1). The median time from injury to presentation was 5 days (IQR, 1-28 days), and the median postpresentation time to surgery was 101 days (IQR, 63-149 days). Insurance status differed significantly by race/ethnicity (χ^2^ = 35.95; *P* < .001). Figure 1 shows the study flow and the 4 analytic domains evaluated.

**Table 1:** Descriptive and Clinical Characteristics of the Analytic Cohort*^*a*^*

Characteristic	Value
Cohort size, n	443
Sex, n (%)	
Female	213 (48.1)
Male	230 (51.9)
Race/ethnicity, n (%)	
Non-Hispanic White	33 (7.4)
Non-Hispanic Black	30 (6.8)
Hispanic	269 (60.7)
Other/unknown	111 (25.1)
Insurance, n (%)	
Private	117 (26.4)
Public (Medi-Cal/Medicaid)	326 (73.6)
Neighborhood deprivation (national COI)	
Median (IQR)	27 (10-63)
Mean ± SD	37.87 ± 31.72
Neighborhood deprivation (health COI)*^*b*^*	
Median (IQR)	48 (32-68)
Mean ± SD	51.4 ± 24.5
Neighborhood deprivation (ADI state decile)*^*c*^*	
Median (IQR)	6 (4-7)
Mean ± SD	5.37 ± 2.36
Age at surgery, y	
Median (IQR)	16.57 (15.58-17.43)
Mean ± SD	16.31 ± 1.67
Time from injury to presentation, days	
Median (IQR)	5 (1-28)
Postpresentation time to surgery, days	
Median (IQR)	101 (63-149)

a ADI, Area Deprivation Index; COI, Childhood Opportunity Index.

b Higher values indicate higher opportunity (less deprivation).

c Higher values indicate greater deprivation.

### Injury-to-Surgery Delay by Insurance

Time from injury to surgery differed significantly by insurance status (Kruskal-Wallis χ^2^ = 40.56; *P* < .001). The median injury-to-surgery time was 81 days (IQR, 53-132 days) for privately insured patients and 130 days (IQR, 95-201 days) for patients with public insurance. Postpresentation delay also differed significantly by insurance (χ^2^ = 38.16; *P* < .001).

### Cartilage Injury (Outerbridge Grade) by Race/Ethnicity

Outerbridge cartilage grade differed by race/ethnicity (χ^2^ = 9.50; *P* = .023). Across the full cohort, grade 0 was present in 323 (72.9%), grade 1 in 9 (2.0%), grade 2 in 93 (21.0%), grade 3 in 13 (2.9%), and grade 4 in 5 (1.1%). When Outerbridge cartilage injury was dichotomized as grades 0 to 2 versus grades 3 to 4, the distribution did not reach significance across race/ethnicity categories (Pearson χ^2^ = 6.07; *P* = .108). The proportion with high-grade injury (grades 3 and 4) was 3.0% (1/33) among non-Hispanic White patients, 10.0% (3/30) among non-Hispanic Black patients, 4.8% (13/266) among Hispanic patients, and 0.9% (1/111) among other/unknown patients. (Of note, cartilage injuries in separate compartments of the knee were counted separately and the total count is higher than the patient population.)

### Time From Injury to Presentation

**Figure 2.**
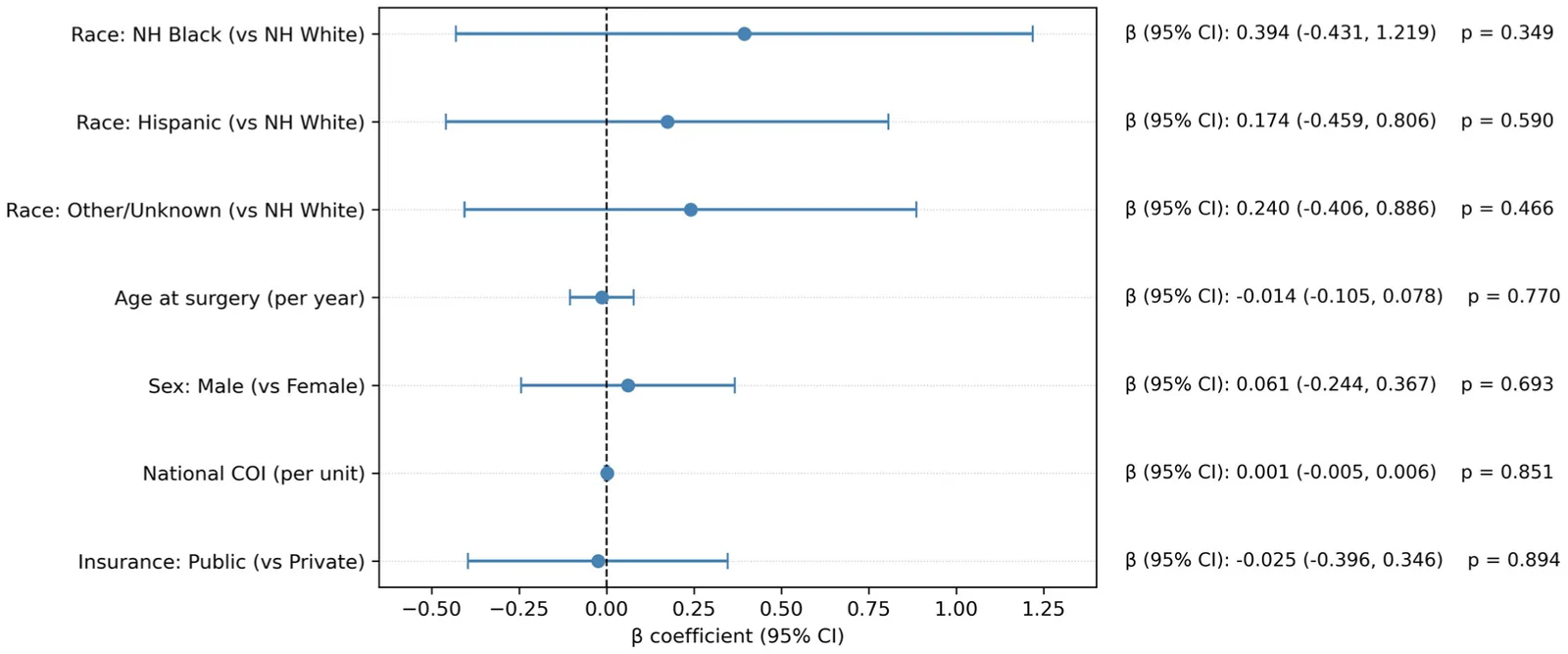
Predictors of time from injury to presentation. Forest plot showing adjusted associations between sociodemographic factors and time from injury to initial clinical presentation, modeled using multivariable linear regression with log-transformed delay as the dependent variable. Estimates are presented as regression coefficients (β) with 95% confidence intervals, with non-Hispanic (NH) White, female sex, and private insurance as reference categories. Models were adjusted for race/ethnicity, age, sex, neighborhood deprivation (national Childhood Opportunity Index [COI] score), and insurance type. *P* values are displayed alongside each covariate.

In linear regression models of log-transformed injury-to-presentation delay, race/ethnicity was not associated with delay in unadjusted analyses (χ^2^ = 0.26; *P* = .968) or after sequential adjustment for age, sex, neighborhood deprivation (national COI: β = 0.001; *P* = .851), and insurance status (public insurance: β = −0.025; *P* = .894). No covariate was independently associated with injury-to-presentation delay in the fully adjusted model (Figure 2).

### Time From Presentation to Surgery

**Figure 3.**
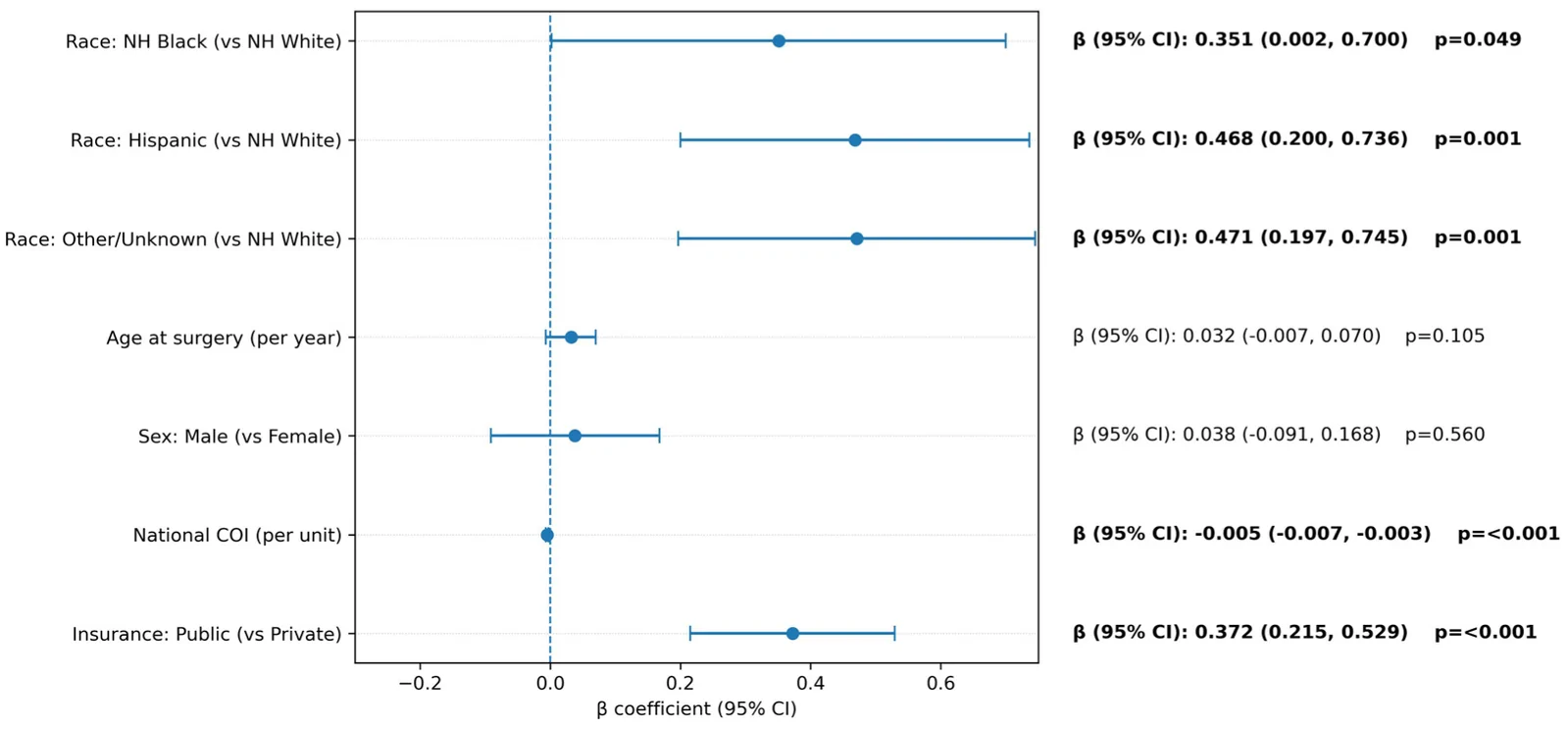
Predictors of time from presentation to surgery. Forest plot showing adjusted associations between sociodemographic factors and time from clinical presentation to surgery, modeled using multivariable linear regression with log-transformed postpresentation delay as the dependent variable. Estimates are presented as regression coefficients (β) with 95% confidence intervals, with non-Hispanic (NH) White, female sex, and private insurance as reference categories. Models were adjusted for race/ethnicity, age, sex, neighborhood opportunity (national Childhood Opportunity Index [COI] score), and insurance type. *P* values are displayed alongside each covariate. This model evaluates the portion of surgical delay occurring after presentation, aligning with the primary equity hypothesis.

Postpresentation time to surgery differed by race/ethnicity (χ^2^ = 26.30; *P* < .001). The median postpresentation delay was 51 days (IQR, 23-91 days) for non-Hispanic White patients, 93 days (IQR, 70-136 days) for non-Hispanic Black patients, 108 days (IQR, 74-157.5 days) for Hispanic patients, and 101 days (IQR, 59-151 days) for other/unknown patients. A lower national COI score was correlated with a longer delay (Spearman ρ = −0.28; *P* < .001). In the fully adjusted linear regression model of log-transformed postpresentation delay using national COI, longer delay remained associated with non-Hispanic Black race/ethnicity (β = 0.351; 95% CI, 0.002-0.700; *P* = .049), Hispanic race/ethnicity (β = 0.468; 95% CI, 0.200-0.736; *P* = .001), and other/unknown race/ethnicity (β = 0.471; 95% CI, 0.197 to 0.745; *P* = .001). Socioeconomic and payer factors were independently associated with longer postpresentation delay, including lower neighborhood opportunity (national COI per unit: β = −0.005; 95% CI, −0.0070 to −0.0025; *P* < .001) and public insurance (β = 0.372; 95% CI, 0.215-0.529; *P* < .001). Older age was not significantly associated with longer delay (β = 0.032; 95% CI, −0.007 to 0.070; *P* = .105), nor was sex (β = 0.038; 95% CI, −0.091 to 0.168; *P* = .560) (Figure 3).

### Sensitivity Analysis Using ADI and Health COI

The 2 continuous delay regressions were refit using the ADI state decile and the health COI subdomain in place of the national COI. Findings were concordant across indices. For time from injury to presentation, neither ADI (β = 0.025; *P* = .474) nor health COI (β = −0.0056; *P* = .099) was significantly associated with delay. For time from presentation to surgery, both ADI (β = 0.045; *P* = .003) and health COI (β = −0.0044; *P* = .003) were each independently associated with longer delay, with non-Hispanic Black, Hispanic, and other/unknown race/ethnicity and public insurance remaining significant in both models (all *P* < .05) (Table 2).

**Table 2:** Sensitivity Analyses for Continuous Delay Outcomes Using ADI and Health COI*^*a*^*

Predictor	ADI β	ADI *P* Value	Health COI β	Health COI *P* Value
Time from injury to presentation				
Race: NH Black vs NH White	0.319	.448	0.322	.437
Race: Hispanic vs NH White	0.116	.712	0.048	.878
Race: other/unknown vs NH White	0.219	.503	0.183	.575
Age at surgery (per year)	−0.014	.766	−0.020	.671
Sex: male vs female	0.044	.776	0.033	.834
Neighborhood deprivation (per unit)*^*b*^*	0.025	.474	−0.0056	.099
Public insurance vs private	−0.059	.756	−0.093	.618
Time from presentation to surgery				
Race: NH Black vs NH White	0.377	**.037**	0.433	**.015**
Race: Hispanic vs NH White	0.559	**<.001**	0.540	**<.001**
Race: other/unknown vs NH White	0.508	**<.001**	0.513	**<.001**
Age at surgery (per year)	0.034	.089	0.033	.093
Sex: male vs female	0.056	.397	0.045	.496
Neighborhood deprivation (per unit)*^*b*^*	0.045	**.003**	−0.0044	**.003**
Public insurance vs private	0.394	**<.001**	0.406	**<.001**

a Multivariable linear regression results with log-transformed delay as the dependent variable, refit using the Area Deprivation Index (ADI) state decile and the health Childhood Opportunity Index (COI) in place of the national COI. All models were adjusted for race/ethnicity, age at surgery, sex, neighborhood deprivation measure, and insurance type, with non-Hispanic White race/ethnicity, female sex, and private insurance as reference categories. Boldface type indicates statistical significance. NH, Non-Hispanic.

b The neighborhood deprivation measure refers to the ADI state decile in the ADI column and the health COI national score in the health COI column; higher ADI values indicate greater deprivation, whereas higher health COI values indicate higher opportunity.

### Probability of Prolonged Postpresentation Delay

Overall, 171 (38.6%) experienced prolonged postpresentation delay >120 days. In adjusted logistic regression using the national COI as the neighborhood covariate, non-Hispanic Black, Hispanic, and other/unknown race/ethnicity were each independently associated with higher odds of prolonged delay relative to non-Hispanic White (OR, 6.02, 6.19, and 7.45, respectively; all *P* < .05). Public insurance was independently associated with higher odds of prolonged delay (OR, 1.80; *P* = .027), and higher neighborhood opportunity was associated with lower odds of prolonged delay (OR, 0.99 per unit COI increase; *P* = .006). Older age was independently associated with higher odds of prolonged delay (OR, 1.19 per year; *P* = .021), whereas sex was not independently associated after adjustment (Table 3).

**Table 3:** Probability of Prolonged Postpresentation Delay (>120 Days) and Associated Factors*^*a*^*

Predictor	OR	Lower 95% CI	Upper 95% CI	*P* Value
Public insurance vs private	1.80	1.07	3.02	.027
National COI (per unit)	0.99	0.98	1.00	**.006**
Race: NH Black vs NH White	6.02	1.17	31.07	**.032**
Race: Hispanic vs NH White	6.19	1.39	27.47	**.017**
Race: Other/unknown vs NH White	7.45	1.65	33.63	**.009**
Age at surgery (per year)	1.19	1.03	1.38	**.021**
Sex: male vs female	1.02	0.67	1.53	.941

a Frequency of prolonged postpresentation delay (>120 days) in the analytic cohort and the results of adjusted logistic regression evaluating associations between descriptive, socioeconomic, and clinical factors and the odds of prolonged delay. Covariates included race/ethnicity, age at surgery, sex, neighborhood opportunity (national Childhood Opportunity Index [COI] score), and insurance type. Boldface type indicates statistical significance. *P* values are 2-sided. NH, Non-Hispanic; OR, odds ratio.

### Delay and Cartilage Injury Severity

When cartilage injury was dichotomized as low-grade versus high-grade, continuous postpresentation delay was independently associated with high-grade cartilage injury after adjustment for race/ethnicity, insurance type, national COI, age, and sex (OR, 1.004 per day; 95% CI, 1.000-1.008; *P* = .048), corresponding to a 57% increase in odds of high-grade injury per 120 additional days of delay (Table 4).

**Table 4:** High-Grade Cartilage Injury (Outerbridge Grade 3-4) by Race/Ethnicity and Association With Delay*^*a*^*

Predictor	OR	Lower 95% CI	Upper 95% CI	*P* Value
Postpresentation delay (per day)	1.004	1.000	1.008	**.048**
Public insurance vs private	0.853	0.245	2.968	.803
National COI (per unit)	0.999	0.980	1.018	.904
Race: NH Black vs NH White	2.64	0.23	30.15	.435
Race: Hispanic vs NH White	1.20	0.13	11.32	.875
Race: other/unknown vs NH White	0.193	0.011	3.514	.267
Age (per year)	1.31	0.88	1.96	.189
Sex: male vs female	1.36	0.50	3.72	.548

a Distribution of cartilage injury severity dichotomized as low-grade versus high-grade with adjusted logistic regression results evaluating associations between prolonged delay and high-grade cartilage injury, controlling for race/ethnicity, age, sex, neighborhood opportunity (national Childhood Opportunity Index [COI] score), and insurance type. Boldface type indicates statistical significance. *P* values are 2-sided. NH, Non-Hispanic; OR, odds ratio.

### Meniscal Injury

Among the analytic cohort, 180 (40.6%) patients had no meniscal tear, 61 (13.8%) had isolated medial tears, 148 (33.4%) had isolated lateral tears, and 54 (12.2%) had both medial and lateral tears. Medial meniscus debridement was performed in 29 (6.5%) patients, while medial meniscus repair was performed in 62 (14.0%). Lateral meniscus repair occurred in 97 patients (21.9%), and lateral meniscus debridement in 96 (21.7%). Meniscal tear prevalence did not differ by race/ethnicity (χ^2^ = 4.27; *P* = .234). In adjusted logistic regression using the national COI, injury-to-surgery delay >120 days was not associated with the presence of any meniscal tear (OR, 0.84; *P* = .395). In this model, race/ethnicity was associated with higher odds of any meniscal tear relative to non-Hispanic White patients, including Hispanic patients (OR, 2.43; *P* = .033) and other/unknown race/ethnicity (OR, 2.48; *P* = .033), and male sex was independently associated with higher odds of any meniscal tear (OR, 1.91; *P* < .001) (Table 5). Using a separate adjusted model with postpresentation prolonged delay (>120 days) as the exposure, prolonged delay was not associated with any meniscal tear (OR, 0.833; *P* = .389). In this model, Hispanic (OR, 2.41; *P* = .034) and other/unknown (OR, 2.48; *P* = .033) race/ethnicity remained associated with higher odds of meniscal tear presence while associations for non-Hispanic Black (OR, 2.31; *P* = .121) did not reach statistical significance; male sex remained independently associated with meniscal tear presence (OR, 1.90; *P* = .001) (Table 6).

**Table 5:** Association Between Injury-to-Surgery Delay and Meniscal Tear Presence*^*a*^*

Predictor	OR	Lower 95% CI	Upper 95% CI	*P* Value
Injury-to-surgery delay >120 days	0.84	0.55	1.26	.395
Public insurance vs private	0.85	0.53	1.38	.515
National COI (per unit)	1.00	0.99	1.01	.948
Race: NH Black vs NH White	2.31	0.80	6.68	.121
Race: Hispanic vs NH White	2.43	1.08	5.48	**.033**
Race: other/unknown vs NH white	2.48	1.08	5.71	**.033**
Age (per year)	0.96	0.85	1.09	.518
Sex: male vs female	1.91	1.29	2.84	**<.001**

a Adjusted logistic regression results evaluating the association between prolonged injury-to-surgery delay (>120 days) and the presence of any meniscal tear. Models control for race/ethnicity, age, sex, neighborhood opportunity (national Childhood Opportunity Index [COI] score), and insurance type. Boldface type indicates statistical significance. *P* values are 2-sided. NH, Non-Hispanic; OR, odds ratio.

**Table 6:** Association Between Postpresentation Delay and Meniscal Tear Presence*^*a*^*

Predictor	OR	Lower 95% CI	Upper 95% CI	*P* Value
Postpresentation delay >120 days	0.833	0.55	1.26	.389
Public insurance vs private	0.848	0.52	1.37	.501
National COI (per unit)	1.000	0.99	1.01	.968
Race: NH Black vs NH White	2.31	0.80	6.68	.121
Race: Hispanic vs NH White	2.41	1.07	5.44	**.034**
Race: other/unknown vs NH White	2.48	1.07	5.70	**.033**
Age (per year)	0.96	0.85	1.09	.527
Sex: male vs female	1.90	1.28	2.82	**.001**

a Adjusted logistic regression results evaluating the association between prolonged postpresentation delay (>120 days), defined as time from initial orthopaedic presentation to surgery, and the presence of any meniscal tear. Models control for race/ethnicity, age, sex, neighborhood opportunity (national Childhood Opportunity Index [COI] score), and insurance type. Boldface type indicates statistical significance. *P* values are 2-sided. NH, Non-Hispanic; OR, odds ratio.

### Subanalysis of Return-to-Sport and Graft Failure Rates Among Social Determinants

**Figure 4.**
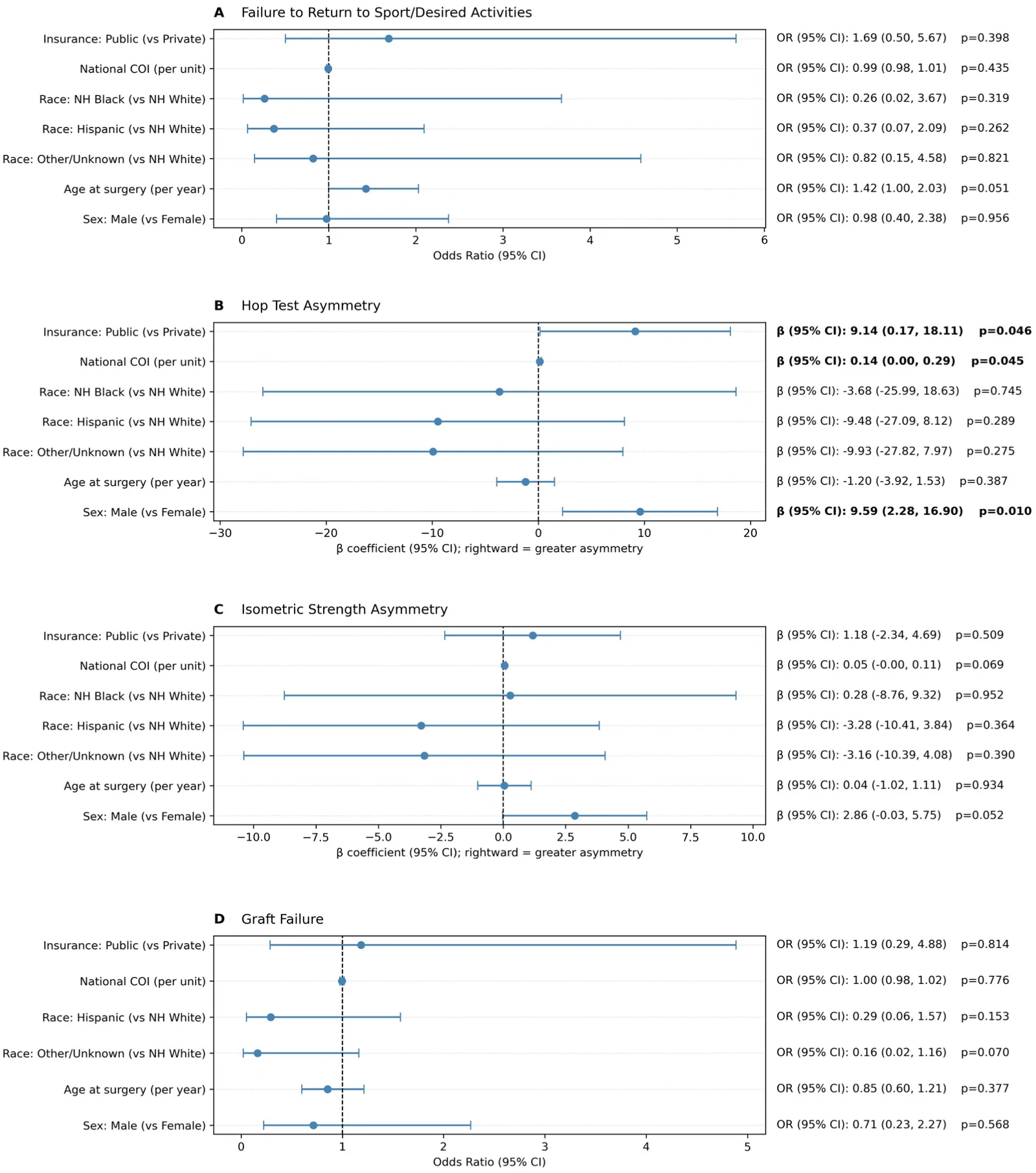
Sociodemographic predictors of return-to-sport outcomes, functional performance, and graft failure. Multipanel forest plot demonstrating adjusted associations between sociodemographic factors and (A) failure to return to sport or desired activities, (B) hop test asymmetry at return to sport, (C) isometric strength asymmetry at return to sport, and (D) graft failure. Panels A and D present odds ratios with 95% confidence intervals derived from multivariable logistic regression models, with failure to return (panel A) and graft failure (panel D) as the dependent variables. Panels B and C present regression coefficients (β) with 95% confidence intervals derived from multivariable linear regression models evaluating hop test and isometric strength asymmetry, respectively. All models were adjusted for race/ethnicity, age at surgery, sex, neighborhood opportunity (national Childhood Opportunity Index [COI] score), and insurance type, with non-Hispanic (NH) White race/ethnicity, female sex, and private insurance serving as reference categories. *P* values are displayed alongside each covariate.

Results of return-to-sport, functional performance, and graft failure analyses are summarized in Figure 4. Among patients with at least 365 days of follow-up (n = 228), both public insurance (OR, 1.69; *P* = .398) and age at surgery (OR, 1.42 per year; *P* = .051) were not independently associated with failure to return to sport (Figure 4A). Among those with functional testing (n = 150), public insurance (β = +9.14; *P* = .046), higher national COI (β = +0.14 per unit; *P* = .045), and male sex (β = +9.59; *P* = .010) were each independently associated with greater hop test asymmetry (Figure 4B). Isometric strength asymmetry showed no independent associations with insurance status or neighborhood opportunity (insurance type and national COI) (Figure 4C). In logistic regression evaluating graft failure (n = 14), neither insurance type nor national COI was independently associated with failure risk. Other/unknown (OR, 0.16; *P* = .070) and Hispanic race/ethnicity (OR, 0.29; *P* = .153) were not statistically associated with lower odds of graft failure (Figure 4D).

## Discussion

In this retrospective single-institution cohort of 443 pediatric patients undergoing ACLR, we found that sociodemographic and structural determinants of health (defined here as insurance type and neighborhood opportunity measured by national/health COI and ADI scores) were strongly associated with delays in operative management, and that these delays were largely attributable to system-level time from orthopaedic presentation to surgery rather than differences in time from injury to presentation. Despite a median injury to presentation time of 5.0 days, the median postpresentation time to surgery exceeded 100 days, and 38.6% of patients experienced a prolonged postpresentation delay >120 days. In adjusted analyses using the national COI as the primary neighborhood deprivation measure, non-Hispanic Black, Hispanic, and other/unknown race/ethnicity; public insurance; lower neighborhood opportunity; and older age were each independently associated with prolonged postpresentation delay, whereas sex was not independently associated after adjustment.

Notably, this cohort is predominantly Hispanic (60.7%) and publicly insured (73.6%), reflecting a demographic composition that differs substantially from much of the prior ACLR literature, in which patients undergoing ACLR are disproportionately White and commercially insured relative to the general population. Our cohort directly addresses this representational gap, providing one of the largest single-institution analyses of pediatric ACLR in a predominantly Hispanic and publicly insured population and, through the structure of our dedicated pediatric orthopaedic urgent care, uniquely enabling isolation of postpresentation system-level delay as the primary driver of disparities in a group that has been systematically underrepresented in the surgical literature.

Prior work has largely focused on injury-to-presentation delay as the primary driver of disparities in ACL care. Baraga et al^1^ noted that patients who were uninsured and those with Medicaid had significantly longer times from injury to diagnosis compared to those with private insurance and were diagnosed 121 versus 56 days after injury. This is in stark contrast to privately insured patients who were diagnosed 14 days after injury.^1^ Our stand-alone pediatric orthopaedic urgent care attempts to address access for patients and decrease evaluation and diagnosis times. In this cohort, the median time to presentation was 5 days, which is significantly shorter than that in the cohort of Baraga et al,^1^ suggesting that families were able to access initial orthopaedic evaluation relatively quickly through our urgent care model. However, substantial inequities emerged in the interval from presentation to surgery. This pattern suggests that disparities in operative care are driven less by delayed injury recognition or delayed orthopaedic care, but stem from system-based delays after initial contact and diagnosis. For instance, insurance approval and acquisition time of MRI studies, surgical authorization, scheduling infrastructure, transportation constraints, or work and school barriers can disproportionately affect families who are socially disadvantaged. In this context, our pediatric orthopaedic urgent care appears to function as a safety net entry point but is not sufficient on its own to eliminate downstream inequities in time to definitive surgical treatment.

Consistent with the structural barriers disproportionately faced by this population, race/ethnicity, insurance status, and neighborhood opportunity were each independently associated with prolonged postpresentation delay in both the binary threshold model (>120 days) (Table 3) and the continuous delay analyses using log-transformed postpresentation delay (Figure 3). These findings have been corroborated across a number of cohorts and systematic reviews and align with prior literature demonstrating disparities in access to timely surgical treatment for ACL injuries.^5,15,16,19^ For instance, Puzzitiello et al^15^ investigated prolonged postpresentation delay in their cohort and found that higher ADI and non-White race are associated with up to a 2.36-fold higher risk of delay to surgery >6 months. In our cohort, race/ethnicity, public insurance, and lower neighborhood opportunity each emerged as independent predictors of prolonged postpresentation delay, underscoring the role of structural and neighborhood-level factors in shaping access to timely operative care after initial orthopaedic evaluation. These findings both align with and extend prior work examining access to care in pediatric ACL populations. For example, Williams et al^19^ demonstrated that publicly insured adolescents experienced substantially longer delays from injury to presentation and had higher rates of chondral injury and meniscal debridement. Together, these data suggest that while insurance status may influence early access and injury severity, downstream delays after presentation may be more strongly driven by broader system-level deprivation factors.

In addition to identifying drivers of delay, we evaluated whether longer delays were associated with worse intra-articular pathology at the time of surgery. We found that continuous postpresentation delay was independently associated with high-grade cartilage injury (Outerbridge grade 3 or 4) after adjustment for race/ethnicity, insurance type, national COI, age, and sex, with each additional day of delay associated with an increased odds of high-grade injury (OR, 1.004 per day; 95% CI, 1.000-1.008; *P* = .048). These findings are supported by prior literature that prolonged time to ACLR is associated with moderate- to high-grade chondral injuries, with similar times from presentation to surgery in socially disadvantaged cohorts.^5,7,11,15^

In contrast, we did not observe an association between prolonged postpresentation delay and the presence of any meniscal tear or pattern of compartmental location. This finding is in direct contrast to prior studies demonstrating that socially disadvantaged patients in respective cohorts have higher rates of irreparable meniscal injuries requiring surgical debridement.^5,15,20^ Given that lateral meniscus tears are common at the time of ACL injury, with medial meniscus tear incidence occurring at higher rates after initial injury, we would have anticipated an association with greater medial meniscus tears in the postpresentation delay cohort.^8,9^ Notably, Hispanic and other/unknown race/ethnicity and male sex were associated with higher odds of any meniscal injury, while non-Hispanic Black race/ethnicity did not reach statistical significance, indicating that injury mechanism, sport participation, and sex-based differences beyond surgical delay may also play a role in the development of concomitant meniscal injuries.

Beyond surgical delay and intra-articular pathology, we also evaluated whether structural determinants (insurance type and neighborhood opportunity) were associated with return-to-sport participation and functional recovery. In secondary analyses restricted to patients with at least 365 days of follow-up, public insurance was not independently associated with failure to return to sport or desired activities. Among the subset who completed functional testing, public insurance and higher national COI were each independently associated with worse hop test asymmetry, whereas no significant associations were observed for isolated isometric strength or graft failure. These findings suggest that structural disadvantage may manifest most strongly in neuromuscular recovery and return-to-play reintegration rather than in intrinsic graft integrity or isolated muscle strength. The associations between public insurance, higher national COI, and worse hop test asymmetry may reflect that public insurance and neighborhood opportunity serve as barriers to consistent physical therapy and rehabilitation, manifesting in greater limb symmetry deficits at the time of return-to-sport testing. However, privately insured patients may disproportionately obtain return-to-sport clearance and functional testing through outside sports medicine physicians or private rehabilitation facilities, meaning those returning for institutional testing may not be representative of the broader privately insured population. Additionally, publicly insured patients who maintain long-term follow-up and complete institutional return-to-sport testing may represent a particularly engaged subset of our cohort. The differential impact on hop symmetry versus isometric strength is physiologically plausible in this pediatric population, as younger patients undergoing ACLR demonstrate less developed neuromuscular control and proprioceptive feedback relative to older adolescents, yet retain the capacity to increase isolated muscle strength through basic exercise. Hop testing demands integrated neuromuscular control and sport-specific movement confidence that is uniquely sensitive to these developmental vulnerabilities, whereas isometric strength may be relatively preserved regardless of rehabilitation access or neuromuscular maturity.^2,4,17^ Future studies prospectively capturing rehabilitation completion and external clearance pathways will be necessary to determine whether structural determinants truly influence neuromuscular recovery after pediatric ACLR.

This study has several limitations. First, the retrospective single-institution design may limit generalizability, particularly because our institution serves as an urban academic quaternary referral center with a pediatric orthopaedic urgent care that may not be widely available in other health systems. Our cohort is predominantly Hispanic and publicly insured, and findings may not be generalizable to the predominantly White and commercially insured populations typically undergoing ACLR at most institutions. These findings are more likely generalizable to inner city safety net hospitals providing pediatric musculoskeletal care to similarly diverse and publicly insured patient populations. Second, time of injury is patient reported and subject to recall bias, and time of first orthopaedic presentation may not capture evaluation outside our system, although unlikely. Third, race/ethnicity and insurance status were derived from the medical record and may be misclassified, and the other/unknown category likely includes heterogeneous populations with distinct access barriers. Fourth, the national COI, ADI, and health COI each reflect neighborhood-level conditions and do not measure individual socioeconomic status, health literacy, or transportation and caregiving constraints that may directly influence scheduling and care completion. Fifth, our cohort was restricted to patients who underwent ACLR and therefore does not capture patients who did not proceed to surgery, sought care elsewhere, or were managed nonoperatively, which may bias estimates of delay and intra-articular pathology. Additionally, the graft failure analysis included only 14 events, and the study was likely underpowered to detect meaningful associations between structural determinants and graft failure risk. Therefore, these results should be interpreted with caution and confirmed in larger cohorts. Finally, cartilage and meniscal pathology were assessed at the time of surgery and documented by different surgeons, which may introduce variability in grading; however, use of standard classifications and dichotomized outcomes mitigates some of this variability.

## Conclusion

In a large cohort of pediatric patients undergoing ACLR at an urban academic quaternary referral center with a stand-alone pediatric orthopaedic urgent care, we found that structural determinants of health were strongly associated with prolonged delays to definitive operative management. Although initial orthopaedic presentation occurred rapidly after injury, disparities emerged primarily in the postpresentation interval, with non-Hispanic Black, Hispanic, and other/unknown race/ethnicity; lower neighborhood opportunity (national COI); and public insurance each independently associated with longer postpresentation delay in both continuous delay and threshold (>120 days) models, with findings remaining significant after sensitivity analyses using ADI and health COI. Importantly, each additional day of postpresentation delay was independently associated with incrementally greater odds of high-grade chondral injury at the time of arthroscopy, underscoring the potential clinical consequences of delayed surgical care. These findings suggest that pediatric orthopaedic urgent care may function as an effective safety net entry point for timely evaluation, but downstream barriers within the health system continue to drive inequities in access to operative treatment. Future efforts should focus on targeted system-level interventions, including streamlined imaging and authorization pathways, patient navigation resources, and expedited surgical scheduling protocols for high-risk populations, to reduce preventable delays and mitigate progression of intra-articular injury in pediatric ACL patients.
